# Benefits of personal music listening for family caregivers of critically ill patients during the post-COVID era

**DOI:** 10.3389/fpsyg.2023.1113269

**Published:** 2023-03-20

**Authors:** Ga Eul Yoo, Sungwon Na, Soo Ji Kim, Jeongmin Kim

**Affiliations:** ^1^Department of Music Therapy, Graduate School, Ewha Womans University, Seoul, Republic of Korea; ^2^Department of Anesthesiology and Pain Medicine, Department of Anesthesia and Pain Research Institute, Yonsei University College of Medicine, Seoul, Republic of Korea; ^3^Music Therapy Education, Graduate School of Education, Ewha Womans University, Seoul, Republic of Korea

**Keywords:** family caregivers, COVID-19 pandemic, intensive care unit, music listening, quality of life, emotional support

## Abstract

**Objective:**

This descriptive study surveyed family caregivers of patients in intensive care units (ICUs) during the COVID-19 pandemic to examine the impact of musical listening on their psychological well-being.

**Method:**

The data collected in this study compared with collected from similar research conducted before the COVID-19 pandemic in 2017. The previous study had 195 participants, and the current study had 92. To measure the participants’ psychological well-being, the Korean version of the Center for Epidemiologic Studies Depression Scale and the World Health Organization Quality of Life Scale were administered. An investigator-constructed questionnaire was also used to collect information related to participants’ engagement in music activities including music listening in their everyday lives and their perceptions of music’s benefits.

**Results:**

A two-way ANOVA showed significant effects for time (e.g., before vs. during COVID-19) and involvement in personal music listening (yes vs. no) on current emotional state, with family caregivers reporting significantly greater negative emotions during COVID-19 than before and personal music listening having a positive effect on perceived emotions. For quality of life there was no significant time effect, while the listening effect was statistically significant, indicating a significantly higher quality of life in the group who engaged in music listening in their everyday lives compared to the group who did not. There were no significant time or listening effects for perceived level of depression.

**Conclusion:**

Given the COVID-19 situation and the need to transition to a post-pandemic era, this study suggests that music listening can be an effective option for family caregivers to implement as a resource for attenuating emotional distress and enhancing self-care.

## 1. Introduction

Since the first outbreak of COVID-19 in 2020, the number of cases increased dramatically with an average of over 1,000 positive COVID-19 cases identified daily during the third wave of the pandemic from November 2020 to February 2021 in Korea (Choi, 2020; Korea Disease Control and Prevention Agency, 2021). The pandemic severely impeded the ability of family caregivers to care their critically ill family members in health care facilities (Irani et al., 2021). Strict intensive care unit (ICU) visitation policies were implemented in 2020. These policies sometimes prohibited all visitors (Suh et al., 2023). These measures remained in place for 3 years until late 2022.

Family caregivers reported feeling increasingly burdened due to the pandemic. In addition to restrictive visitation policies, the high rate of infection, unpredictable symptom development, and widespread anxiety in the community all negatively impacted family caregiver well-being (Rahimi et al., 2021). As a result, restrictive visitation policies it was more challenging for family caregivers to tend to their ill family members in ICUs (Andrist et al., 2020; Valley et al., 2020), and this resulted in increased frustration and anger (Hamilton et al., 2020; Morley, 2020). Access to social support from medical and community sources also became more difficult (Azoulay et al., 2020; Beach et al., 2021; Hwang et al., 2021). The COVID-19 pandemic has had multiple impacts on family caregivers, including exacerbating physical, psychological, emotional, and social stressors (Azoulay et al., 2020). However, different emotional distress-related symptoms need to be understood and addressed independently, such as anxiety or fear as a proactive response to uncertainty or situational threats and depression as an internalized reaction to lack of control or loss (Gruber et al., 2021). Research has found that anxious responses are accompanied by heightened arousal to emotional stimuli, regardless of the specific emotion, while depressive responses are associated with a failure to sustain a positive response to pleasant stimuli (Larson et al., 2007). These findings emphasize the importance of considering multiple factors as affecting the psychological health of family caregivers.

Previous studies have shown the potential benefits of incorporating music-related activities including music therapy, in addressing the impact of COVID-19 on family caregivers. Research has demonstrated the effectiveness of music in alleviating emotional distress and empowering family caregivers through enhanced self-care (Jung and Na, 2019; Yoo et al., 2021). The COVID-19 pandemic has highlighted the need for additional support strategies, especially during times of crisis when traditional sources of support may be inaccessible. Music can be an easily accessible and affordable means for self-regulation when access to other activities is limited due to lockdowns or social distancing (Cabedo-Mas et al., 2020; Ziv and Hollander-Shabtai, 2022). Personal music activity, which can be performed independently by an individual in their home, holds particular promise for promoting psychological and emotional stability (e.g., decreased depression and improved quality of life) and has been documented as positively impacting diverse populations in various countries (Cabedo-Mas et al., 2020; Krause et al., 2020; Roese and Merrill, 2021). With COVID-19 interfering with people’s ability to engage in music activities that require social contact (e.g., singing together or attending concerts), personal use of music at home, and especially listening to music, has increased with the greater availability of music through various online platforms (Fink et al., 2021; Ziv and Hollander-Shabtai, 2022).

Given the potential benefits of music activities for family caregivers of ICU patients, our previous study (Yoo et al., 2021) investigated family caregivers’ engagement in music activities prior to the COVID-19 pandemic and found that active involvement in singing alleviated their emotional distress and served as a self-care strategy for emotional regulation. The current study aimed to extend the previous research by comparing data from both studies (before the pandemic and during the pandemic) to investigate the impact of the pandemic on the psychological and emotional well-being of family caregivers of ICU patients and the impact of personal music engagement on their persistent feelings such as depression and quality of life, as well as their current emotional reactions (e.g., happiness, sadness, anger, fear, and comfort) before and after the emergence of COVID-19. Therefore, the purpose of this study was to investigate whether personal engagement in music activities differentially impacted psychological and emotional states of family caregivers of ICU patients before and during the pandemic.

## 2. Materials and methods

### 2.1. Study design and participants

This study is a descriptive study that surveyed family caregivers of ICU patients. The study examined the psychological and emotional states of these caregivers, including their level of depression, quality of life, and current emotional states, and investigated whether they engaged in personal music activities. Furthermore, this study compared the measured variables with data from before the COVID-19 pandemic to investigate differences in family caregivers’ psychological and emotional states before and during COVID-19, and to examine how these states varied depending on whether the caregivers engaged in personal music activities. The pre-pandemic data used in this study were collected from January 2017 to July 2017 as part of our previous study (Yoo et al., 2021). For data during the COVID-19 pandemic, new participants were surveyed in 2021.

All procedures were approved by the Institutional Review Board of Severance Hospital (4-2020-1,460) in Seoul, Korea. Inclusion and exclusion criteria for participants were consistent across both studies. A detailed description of the pre-pandemic recruitment procedures and sample can be found in our previous study (Yoo et al., 2021). In both the pre-pandemic and pandemic studies, the participants were adults aged 18 years or older who had a family member admitted to a surgical ICU (SICU). Among family caregivers, only direct family members (i.e., spouse, parent, adult child) who were responsible for providing primary care (e.g., providing day-to-day care and making first-line medical decisions about treatment) for the patient were included. Caregivers who were not direct members (e.g., siblings) or those who did not provide primary care were excluded. The sample size for this study was calculated using G power*3.1 (Faul et al., 2007) based on the effect size from a previous study (Yoo et al., 2021), with a desired power of 0,95, and an alpha level of 0.05. The minimum sample of 285 participants for two groups was determined to be sufficient for this study.

### 2.2. Measures

In this study, measures of psychological and emotional well-being such as depression, quality of life, and current emotional states, as well as engagement in personal music activity were used. To measure depression, the Center for Epidemiologic Studies Depression Scale (CES-D; Radloff, 1991) was administered. The Korean version of the CES-D (Shin et al., 1991) consists of 20 items rated on a 4-point Likert scale corresponding to how often the participant felt the way presented in each item over the past week. The Korean version of the CES-D has been validated and the cutoff for the scale is 16 with a score greater than 16 indicating risk for depression. To assess quality of life in caregivers of ICU patients, the abbreviated World Health Organization Quality of Life Scale [WHOQOL-BREF; 25 (World Health Organization, 1998)] was administered. The Korean version of the WHOQOL-BREF (Min et al., 2002) consists of 26 items rated on a 5-point Likert scale based on evaluations of four domains: physical health, psychological health, social relationships, and environment. The Korean version was validated with a total raw score ranging from 26 to 130, and the score for each domain ranges from 4 to 20, with a higher score representing a higher level of perceived quality of life.

Furthermore, an investigator-constructed questionnaire was used to collect information about each participant’s demographics, their engagement in personal music activities, and their perceptions on the benefits of music use. The items included whether each participant had engaged in each of three music activities (i.e., music listening, singing, and instrument playing) in their everyday lives after their family member became sick and what benefits they believed this involvement with music brought to their lives (e.g., physically, cognitively, emotionally, socially, and environmentally). Participants self-administered the questionnaire to provide personal information. In the questionnaire, a 100 mm visual analogue scale was incorporated for the participants to self-rate their current emotional states. Participants were instructed to rate the degree to which they experienced the presented emotion (i.e., happiness, sadness, anger, fear, and comfort) in the past week on a straight line anchored at the ends by “feeling the presented emotion not at all” and “feeling the emotion very much.”

### 2.3. Procedures

Data collection was conducted between February 2021 and July 2021 during a period of highly restrictive hospital visitation policies. Participants were recruited through posters placed in the waiting areas of the SICU units and eligible participants were those who approached the research team after reading the poster. Each participant provided written informed consent prior to their participation in the study. Participants then completed a self-administered questionnaire in a private and quiet area of the hospital typically used for counseling.

### 2.4. Data analysis

The data collected in 2017 were compared with the data collected in 2021. An independent t test was conducted to assess differences in psychological and emotional measures as well as demographic information (i.e., participant’s age, patient’s age, days on mechanical ventilation, and number of ICU stays) between the two groups. A chi-square test was used to investigate if the two groups differed in terms of whether they engaged in music activities after they became a family caregiver. Furthermore, a two-way ANOVA was conducted to determine if there were significant differences in the measured assessment (i.e., WHOQOL-BREF and CES-D) depending on the time point (before COVID-19 sample and during COVID-19 sample) and engagement in music listening (i.e., yes and no). Finally, among the participants who perceived music as beneficial, the percentage who were currently engaging in music listening at the time of data collection versus the percentage who were not was compared by conducting a chi-square test.

## 3. Results

For this study conducted in 2021, a total of 104 family caregivers were initially surveyed. Five surveys were excluded due to incomplete responses, and additional seven participants were excluded because they did not meet the inclusion criteria of being a direct family member. The final sample included 92 caregivers with a mean age of 47.3 years. The comparison data from 2017 had a sample of 195 caregivers with a mean age of 51.2 years (Krause et al., 2020) was also analyzed. Detailed information about the 2017 sample can be found in our previous study (Krause et al., 2020). The 2021 sample included 38 (41.3%) spouses, 43 (46.7%) adult children, and 11 (12.0%) parents of ICU patients. The participants’ ill family members (i.e., ICU patients) had been in the ICU for 3.8 days on average. Demographic information about the participants from the study conducted before COVID-19 and the study conducted during COVID-19 is summarized in Table 1. When the two groups were compared, significant differences were found in terms of the patient’s age (*p* < 0.001), duration of mechanical ventilation (*p* < 0.001), and length of ICU stay (*p* < 0.001). The sample from 2021 showed a significantly younger patient age, longer duration of mechanical ventilation and longer ICU stays compared to the sample from 2017.

**Table 1:** Participants’ demographic information.

Variable	Before COVID-19 (2017 sample) (*N* = 195)	During COVID-19 (2021 sample) (*N* = 92)	*t*/*χ*^2^	*p*
Sex (male: female)	69: 126	38: 54	0.94	0.361
Age (years), *M* ± *SD*	51.2 ± 13.9	47.2 ± 13.9	2.24	0.026*
Relationship to patient			6.24	0.056
Spouse	86 (44.1%)	38 (41.3%)		
Adult child	98 (50.3%)	43 (46.7%)		
Parent	11 (5.6%)	11 (12.0%)		
Patient information, *M* ± *SD*				
Age of patient (years)	65.3 ± 13.3	57.6 ± 15.5	4.39	<0.001***
Duration since onset of diagnosis (months)	28.1 ± 72.6	25.4 ± 40.0	0.34	0.733
Diagnosis at ICU admission			244.62	<0.001***
Elective surgery	170 (87.2%)	75 (81.5%)		
Emergent surgery	15 (7.7%)	6 (6.5%)		
Respiratory failure	4 (2.1%)	1 (1.1%)		
Cardiac failure	2 (1.0%)	1 (1.1%)		
Sepsis	3 (1.5%)	7 (7.6%)		
Acute bleeding	0 (0.0%)	2 (2.2%)		
Acute kidney injury	1 (0.5%)	0 (0.0%)		
APACHE II score (0–75)	17.7 ± 7.2	17.2 ± 7.6	0.51	0.607
Length of mechanical ventilation (days)	1.0 ± 2.3	2.1 ± 2.9	−3.33	<0.001***
Length of ICU stay (days)	2.2 ± 1.1	3.8 ± 4.1	−5.22	<0.001***

APACHE, Acute Physiology and Chronic Health Evaluation. *p < 0.05. **p < 0.01. ***p < 0.001.

### 3.1. Differences in psychological and emotional measures between 2017 and 2021 samples

The descriptive statistics for the psychological measures of the participants are displayed in Table 2. To control for the effects of the aforementioned differences between the two sample, a one-way ANCOVA was conducted, while controlling for the participant’s age, the patient’s Acute Physiology and Chronic Health Evaluation II (APACHE II) score, and the patient’s length of mechanical ventilation, and length of ICU stay. Perceived level of depression and quality of life were not significantly different depending on the time point. When examining the effects of the covariates, it was found that age was a significant factor in the effect of the time point on the measured quality of life, *F*(1, 286) = 21.175, *p* < 0.001, while both the age of participants and the number of ICU days experienced by patients were significant factors in the effect of the time point on perceived depression, *F*(1, 286) = 4.072, *p* = 0.045 (for the effect of age) and *F*(1, 286) = 10.140, *p* = 0.002 (for the effect of ICU days). In addition, all emotions except for anger were significantly different between the two groups, with those during COVID-19 reporting significantly higher levels of sadness and fear and significantly lower levels of happiness and comfort. For controlled covariates, the participant’s age was found to significantly affect the difference in their happiness levels between the two time points, *F*(1, 286) = 7.223, *p* = 0.008, and anger, *F*(1, 286) = 9.226, *p* = 0.003, and the length of patient’s ICU stay significantly affected the effect of the time point on the sadness felt by caregivers, *F*(1, 286) = 4.051, *p* = 0.045.

**Table 2:** Subjective measures of mood and well-being by family caregivers of ICU patients.

Variable	Before COVID-19 (2017 sample) (*N* = 195)	During COVID-19 (2021 sample) (*N* = 92)	*t*	*p*
CES-D	16.7 ± 10.6	15.4 ± 11.8	3.383	0.067
WHOQOL-BREF	88.1 ± 15.1	88.9 ± 13.5	0.171	0.679
Emotional state				
Happiness	51.6 ± 25.3	44.5 ± 24.7	4.860	0.028*
Sadness	44.9 ± 29.5	55.9 ± 29.5	4.745	0.030*
Anger	37.3 ± 26.3	36.8 ± 26.4	0.000	0.999
Anxiety	42.3 ± 53.9	53.8 ± 30.4	5.603	0.019*
Comfort	46.7 ± 26.2	39.1 ± 26.9	4.062	0.045*

A one-way ANCOVA was conducted controlling for the participant’s age, the patient’s length of ventilation, and length of ICU stay. WHOQOL-BREF, World Health Organization Quality of Life Scale abbreviated version; CES-D, Center for Epidemiologic Studies Depression Scale. *p < 0.05. **p < 0.01.

### 3.2. Differences in levels of personal music engagement between 2017 and 2021 samples

In the 2021 sample, 60.9% of participants were currently engaging in music listening, 12.0% in singing, and 4.3% in instrument playing. The percentage of these participants who engaged in music listening was higher (51.3% in the 2017 sample) and their engagement in other types of music activities (i.e., singing and instrument playing) was lower (11.3% for singing and 5.1% for instrument playing in the 2017 sample) when compared with 2017 sample. The percentage of participants who reported that they continued to sing and play an instrument after their family member became ill was similar in both samples (11.3% in the 2017 sample and 11.0% in the 2021 sample for singing; 5.1% in the 2017 sample and 4.4% in the 2021 sample for instrument playing). While 51.3% of participants maintained their engagement in music listening after their family member became ill in the 2017 sample, a greater percentage of participants (60.9%) reported continuing to engage in music listening in the 2021 sample.

When asked if music use benefits their health, 72.8% of the participants in the 2021 sample reported yes, while 88.2% of the 2017 sample reported yes. A chi-square test indicated that the percentage of participants who perceived music as beneficial significantly decreased during COVID-19 compared to before COVID-19 (*χ*^2^ = 21.230, *p* = 0.020). When asked about the area in which engagement in music activities would have a beneficial impact, the emotional domain was reported the most frequently at both time points (78.5% in the 2017 sample and 88.2% in the 2021 sample). A chi-square test showed no significant relationship between when data were collected (i.e., before and during COVID-19) and the domain in which music was perceived to be beneficial (*χ*^2^ = 8.412, *p* = 0.999).

Furthermore, it was investigated whether engagement in music activities affected the participants’ perceived psychosocial health (i.e., quality of life and level of depression) and what benefits family caregivers of ICU patients identified as resulting from their music activities. Among the three types of music activities, whether participants currently engaged in music listening was analyzed as a factor for influencing psychosocial health of participants given that only 12.0% of respondents reported having recent singing experience and only 4.4% of respondents reported having recent instrument playing experience. Since these percentages were not suitable for subgroup analysis, further analyses were conducted to identify differences in the psychological measures depending on engagement in music listening.

### 3.3. Effects of time point and engagement in music listening on psychological and emotional measures between 2017 and 2021 samples

A two-way ANOVA was conducted to compare the psychological health of family caregivers of ICU patients depending on the time point (i.e., before COVID-19 sample and during COVID-19 sample) and current engagement in music listening (i.e., yes and no). At both time points, the listening group reported a lower level of depression and higher level of quality of life compared to the nonlistening group. Also, the listening group at both time points reported a greater degree of happiness and comfort and a lower degree of anger, sadness, and fear than the nonlistening group (see Table 3).

**Table 3:** Perceived levels of depression and quality of life for the two samples.

Variable	Before COVID-19		During COVID-19	
	Listening (*n* = 100)	Nonlistening (*n* = 95)	Listening (*n* = 56)	Nonlistening (*n* = 36)
CES-D	15.2 (10.1)	18.3 (11.0)	14.8 (10.9)	16.4 (13.3)
WHOQOL	90.7 (15.7)	85.4 (14.0)	90.0 (14.4)	87.3 (11.9)
Emotional state				
Happiness	56.8 (25.2)	46.1 (24.3)	47.1 (25.9)	40.4 (22.3)
Sadness	38.9 (27.1)	51.2 (30.7)	51.5 (29.7)	62.6 (28.2)
Anger	34.1 (25.9)	40.8 (26.4)	34.9 (27.3)	39.8 (25.0)
Fear	37.1 (27.9)	47.7 (29.3)	50.1 (30.2)	59.4 (30.2)
Comfort	50.7 (25.5)	42.4 (26.4)	42.6 (27.7)	33.8 (25.0)

Values in the table are *n*(%).

**Figure 1:**
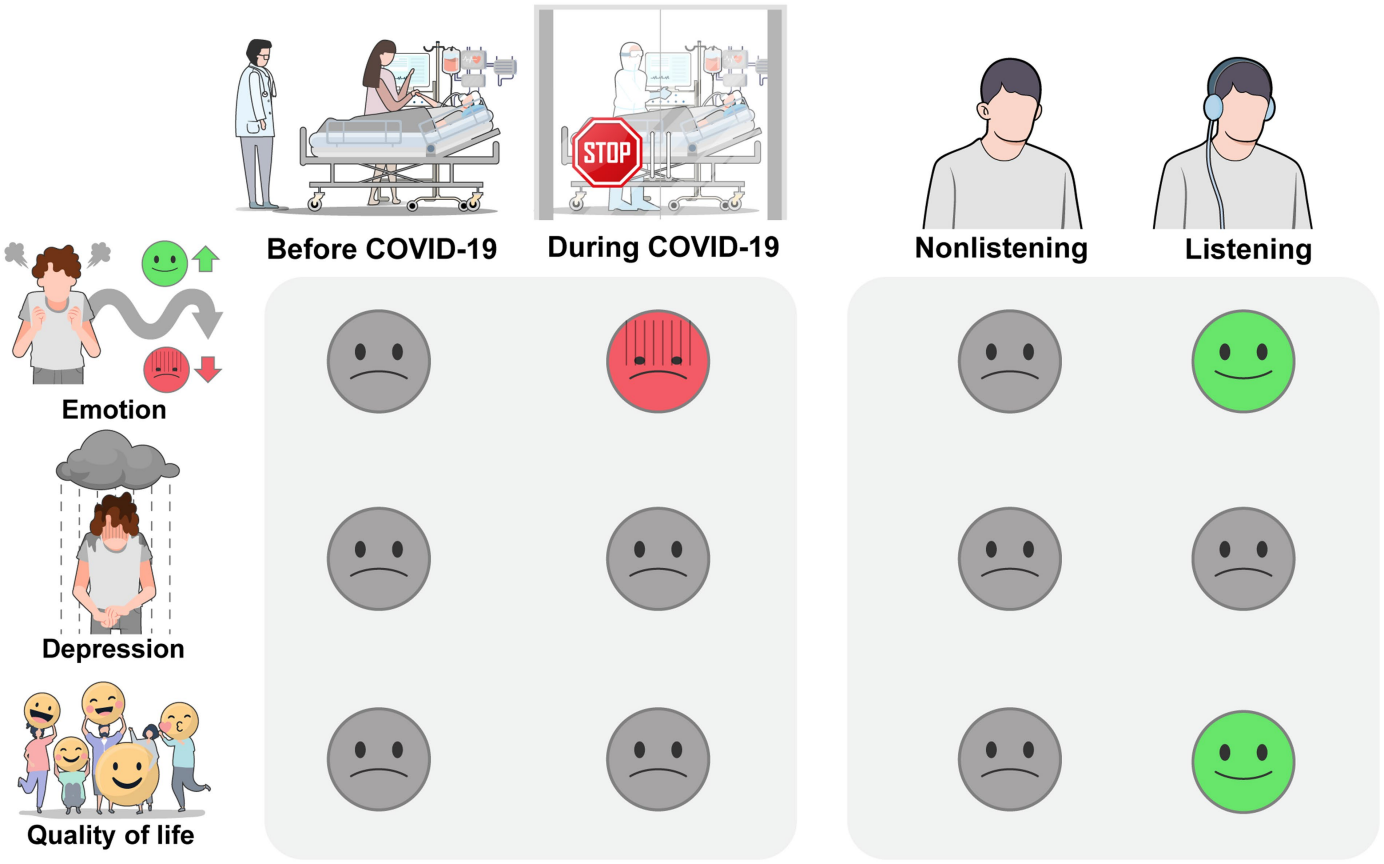
Self-reported psychological well-being of family caregivers of ICU patients before and during the COVID-19 pandemic and whether currently engaging in music listening. During COVID-19, the emotional states as an acute response to current stressors were significantly more negative, and current engagement in music listening showed a significant change in the opposite direction. For perceived depression as a more chronic mood state, time point and current engagement in music listening did not result in significant changes. For quality of life, which is a general perception of life influenced by multiple factors, there was no significant difference between the two samples. However, the listening group showed a significantly higher level of quality of life compared to the nonlistening group.

The results of this two-way ANOVA are displayed in Table 4. With regard to perceived level of depression, two-way ANOVA results showed that there were no significant time or listening effects. No significant interaction effect between time and listening was found. For quality of life, there was no significant time effect, while the listening effect was statistically significant, indicating a significantly higher level of quality of life in the listening group compared to the nonlistening group (see Figure 1). Also, no significant interaction effect between time and listening was found. In terms of rated emotional states, significant time and listening effects were found for all the emotions except anger. With regard to the time effect, during COVID-19, a significantly lower degree of happiness and comfort and significantly greater degree of sadness and fear were found compared to before COVID-19. The same trend was found in the listening group compared to the nonlistening group, exhibiting a significantly greater level of positive emotions and significantly lower level of negative emotions except anger (see Figure 1). No significant interaction effects between time point and listening were found for any of the emotions, indicating differences in ratings on the emotional states depended on the emotion type and were similar before and during COVID-19.

**Table 4:** Results of the two-way ANOVA for the effects of time point (before and during COVID-19) and music listening (yes vs. no).

Variable	Time point (before and during COVID-19)		Music listening		Time point * Music listening	
	*F*	*p*	*F*	*p*	*F*	*p*
CES-D	0.631	0.428	3.331	0.069	0.262	0.609
WHOQOL	0.088	0.767	4.379	0.037*	0.769	0.381
Emotional state						
Happiness	5.008	0.026*	8.009	0.005**	0.442	0.507
Sadness	8.799	0.003**	7.435	0.007**	0.445	0.505
Anger	0.021	0.886	1.721	0.191	0.551	0.458
Fear	11.199	0.001**	6.152	0.014*	0.197	0.658
Comfort	6.782	0.010*	6.595	0.011*	0.000	0.988

*p < 0.05. **p < 0.01.

Among the participants who perceived music as beneficial, the ratio of participants who were currently listening to music versus those who were not at the time of data collection differed before and during COVID-19. There was a significant relationship between current engagement in music listening and time point (*χ*^2^ = 4.091, *p* = 0.043), and it was found that a greater percentage of participants currently was engaging in music listening during COVID-19 (67.2%) than before COVID-19 (51.2%).

## 4. Discussion

In this study, we investigated the perceptions of family caregivers of ICU patients who were hospitalized during the COVID-19 pandemic. Family caregivers’ level of depression and quality of life were measured, and their engagement in music activities and its benefits were assessed. In addition, we compared the results of this study to data from a similar sample from before the COVID-19 pandemic.

First, before and during COVID-19, the mean score of CES-D for family caregivers of ICU patients was close to or just above the cutoff point for clinical depression. This aligns with the finding that family caregivers experience extensive psychosocial burdens and emotional distress (Pochard et al., 2005; Choi et al., 2012; Alfheim et al., 2019) and gives rise to the need for interventions that target caregivers’ psychological well-being. When comparing the caregivers’ perceptions on their psychological well-being before and during COVID-19, there were no significant differences between the time points in terms of perceived depression and quality of life, while ratings on current emotional states were significantly different between the two groups. During COVID-19, caregivers reported feeling significantly higher levels of sadness and fear and lower levels of happiness and comfort compared to the sample measured before COVID-19. Interestingly, no significant differences in perceived depression and quality of life were found between the two time points. Although previous studies have demonstrated that the pandemic aggravated people’s depression and decreased their quality of life in general (Azoulay et al., 2020; Irani et al., 2021; Rahimi et al., 2021), such an effect was not significant among the family caregivers of ICU patients in this study. This finding may be attributed to the fact that family caregivers of ICU patients already have a high level of depression and a low level of quality of life, and such distress should be understood as a fundamental issue of family caregivers of ICU patients regardless of the other situations that may impact them, such as the COVID-19 pandemic.

Meanwhile, when comparing the reported current emotional states as an immediate reaction to situational stressors, significant differences were found between the two time points. This result can be explained by the fact that mood as a more persistent feeling (e.g., depression) is separate from emotion as a relatively more temporary reaction to current environmental stressors (Larson et al., 2007). The COVID-19 pandemic caused severe disruptions forcaregiving environment for family caregivers of ICU patients, such as limiting access to caregiver resources (e.g., restricting visits with patients and contact with medical staff), and it might have exacerbated family caregivers’ acute emotional distress (Hamilton et al., 2020; Hwang et al., 2021). Given that persistence in negative emotion leads to more chronic mood states and lower perceived quality of life (Gendolla and Brinkmann, 2005), the current study’s results support the importance of timely and appropriate interventions targeting such negative emotions.

During COVID-19, the participants frequently engaged in music listening, while participation in more active forms of music activity, such as singing and playing an instrument, declined compared to the sample measured prior to the pandemic. This change in music engagement can be attributed to the widespread accessibility and affordability of music listening, even under environmental restrictions that affect human activities (Roese and Merrill, 2021; Ziv and Hollander-Shabtai, 2022). This also supports that music listening can be an adaptive and protective strategy for promoting psychological health while minimizing the risk of widespread infection (Mas-Herrero et al., 2020). Given the growing need to address the emotional and social stressors faced by caregivers during crises, such as the ongoing pandemic, music listening can be considered a feasible strategy for self-care and self-regulation. However, previous studies identified more active forms of music activity (e.g., singing and instrument playing) as being more effective coping strategies since more active control of their actions during such music activity empowers the family caregivers more effectively (Jung and Na, 2019; Yoo et al., 2021). Future studies will need to investigate how music listening can be incorporated into family-centered care in more diversified ways while requiring more active and creative engagement.

Finally, this study investigated the impact of personal music listening on the perceived psychological and emotional well-being of family caregivers of ICU patients before and during the pandemic (see Figure 1). Results showed that during the pandemic, there were significant increases in negative emotions, such as sadness, and decreases in positive emotions, such as happiness. However, engagement in personal music listening had a positive effect on emotional states and quality of life. These results suggest that while the COVID-19 pandemic may trigger an immediate reaction to environmental stressors, listening to music can help mitigate these impacts. The lack of an effect on depression levels indicates that engagement in music listening, which is a relatively passive activity, may not be sufficient to address the chronic and clinical symptoms of depression. This also corroborates that more active engagement in music, such as singing, or a more structured intervention may be necessary to effectively alleviate depression.

When examining the caregivers’ perceptions on the benefits of music, they were less positive during COVID-19 compared to the sample measured before COVID-19; however, among the caregivers who reported that music was beneficial for their health, a higher percentage of respondents were currently engaging in music listening during COVID-19 than before the pandemic. This decrease in the perceived benefits of music may be attributed to the limited options for music activities available to family caregivers during COVID-19. Despite these restrictions, engagement in music listening improved the caregivers’ perceptions not only of their psychological and emotional well-being but also of the benefits of music to a greater extent than the nonlistening group. This suggests that utilizing resources for self-regulation, such as music, can effectively mitigate the emotional distress (Shaffer et al., 2016; Ferreri et al., 2021) resulting from caregiving responsibilities and exacerbated by physical and psychological restrictions during the COVID-19 pandemic. Engagement in music listening could also help caregivers identify meaningful and rewarding aspects in their immediate environment as seen in enhanced perception on their quality of life. And for that emotional resource, engagement in music listening can be one readily available, effective, and creative medium for increasing arousal and motivation to a desirable level (Jung and Na, 2019; Yoo et al., 2021) and for empowering family caregivers to better cope with their situation and gain a greater sense of control (Wartella et al., 2009; Ferreri et al., 2021).

Given the COVID-19 situation and the need to transition to a post pandemic era, this study suggests that music listening can be an effective option for family caregivers to implement as a resource for self-care, although its effects would not lead to significant changes in depression, as more chronic and clinical symptoms require a more systematic intervention. Considering the uncontrolled nature of ICU admission, the collection of data from two different groups resulted in differences in terms of patient age and duration of ICU stays. These differences limit the generalizability of the results and constrain their applicability to a generalized population. Perceived changes in the caregiving burden and corresponding emotional distress in family caregivers of critically ill patients with chronic diseases before and after the COVID-19 pandemic should also be further investigated. Furthermore, in this study, engagement in music listening was examined as a mediating factor for influencing the psychological health of caregivers, and the effects of singing and instrument playing were not examined due to the unbalanced percentage of participants who engaged in these music activities. This limits the explanation of differential effects of passive versus active forms of music engagement on family caregivers of ICU patients. Further studies are needed to directly compare engagement in passive and active music activities and to identify how each activity differs in supporting the needs of caregivers during the COVID-19 pandemic and post-COVID era.

## 5. Conclusion

In conclusion, this study demonstrates the impact of the COVID-pandemic and personal music activities on the psychological and emotional well-being of family caregivers of ICU patients. The results show that family caregivers experienced significant decreases in happiness and comfort and increases in sadness and fear during the pandemic. However, engaging in personal music listening was found to have a reverse effect, increasing positive emotions and decreasing negative emotions, as well as improving quality of life. The measure of depression did not vary significantly between the time points and engagement in music listening. These results indicate the importance of considering the multiple factors that influence persistent emotions, such as depression. It is important to note that personal music engagement may not have significant effects on depression, which requires more intensive and clinical intervention from professionals, such as music therapists. In light of the ongoing impact of the COVID-19 pandemic, self-initiated music engagement, such as personal music listening, should be encouraged as a form of self-care in the post-COVID era. At the same time, it is important to acknowledge the limitations of such approaches and to continue to develop and expand systematic and intensive music therapy interventions to address more complex emotional and clinical issues faced by this population of family caregivers of ICU patients.

## Data availability statement

The original contributions presented in the study are included in the article/supplementary material, further inquiries can be directed to the corresponding authors.

## Ethics statement

The studies involving human participants were reviewed and approved by the Institutional Review Board of Severance Hospital (4-2020-1460). The participants provided their written informed consent to participate in this study.

## Author contributions

GY: formal analysis and writing. SN: conceptualization and supervision. SK: supervision and validation. JK: conceptualization and investigation. All authors contributed to the article and approved the submitted version.

## Conflict of interest

The authors declare that the research was conducted in the absence of any commercial or financial relationships that could be construed as a potential conflict of interest.

## Publisher’s note

All claims expressed in this article are solely those of the authors and do not necessarily represent those of their affiliated organizations, or those of the publisher, the editors and the reviewers. Any product that may be evaluated in this article, or claim that may be made by its manufacturer, is not guaranteed or endorsed by the publisher.
